# Form follows function in Triticeae inflorescences

**DOI:** 10.1270/jsbbs.22085

**Published:** 2023-02-14

**Authors:** Shun Sakuma, Ravi Koppolu

**Affiliations:** 1 Faculty of Agriculture, Tottori University, Tottori 680-8553, Japan; 2 Research Group Plant Architecture, Leibniz Institute of Plant Genetics and Crop Plant Research (IPK), Corrensstr. 3, OT Gatersleben, 06466 Seeland, Germany

**Keywords:** inflorescence, Triticeae, fertility, spike branching, spikelet development, dryland, high temperature

## Abstract

Grass inflorescences produce grains, which are directly connected to our food. In grass crops, yields are mainly affected by grain number and weight; thus, understanding inflorescence shape is crucially important for cereal crop breeding. In the last two decades, several key genes controlling inflorescence shape have been elucidated, thanks to the availability of rich genetic resources and powerful genomics tools. In this review, we focus on the inflorescence architecture of Triticeae species, including the major cereal crops wheat and barley. We summarize recent advances in our understanding of the genetic basis of spike branching, and spikelet and floret development in the Triticeae. Considering our changing climate and its impacts on cereal crop yields, we also discuss the future orientation of research.

## Introduction

The grass tribe Triticeae is of critical importance to the global food supply. It includes major cereal crops, such as bread wheat (*Triticum aestivum*), pasta wheat (*T. turgidum* ssp. *durum*), and barley (*Hordeum vulgare*), as well as climate-resilient crops such as rye (*Secale cereale*) and triticale (×*Triticosecale*). In addition to these cereals, the Triticeae comprises about 350 other species, including the economically significant perennial fodder grasses *Agropyron*, *Elymus*, *Leymus*, and *Psathyrostachys* (Barkworth and Bothmer 2009). The annual cereal plant species are most abundant in western Asia and around the Mediterranean region, but are also found in temperate and semi-arid regions around the world.

**Fig. 1.**
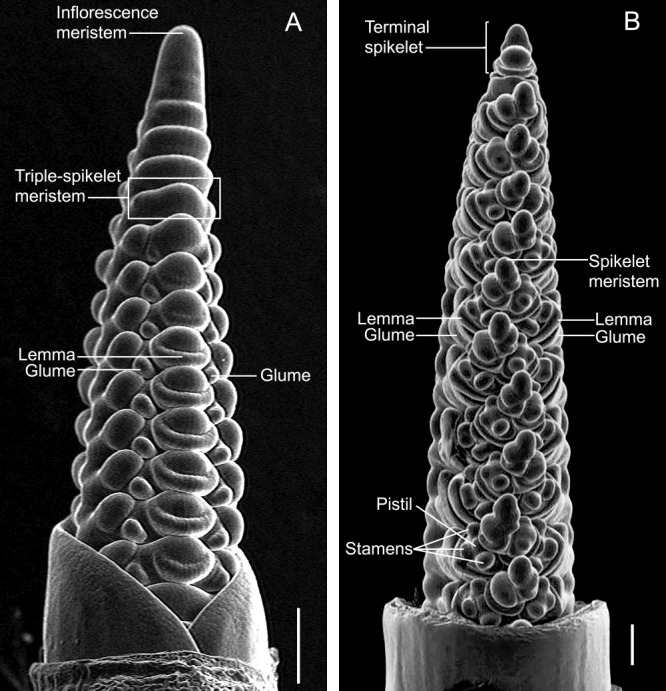
Inflorescence structure of barley and wheat. Scanning electron microscopy images of immature barley (A) and wheat (B) spikes are shown. The determinacy of the inflorescence meristem is lost in barley, whereas it is maintained to differentiate a terminal spikelet in wheat. The position of glumes is different. The barley triple-spikelet meristem is a determinate, and the wheat spikelet meristem is indeterminate. Scale bars = 200 μm.

**Fig. 2.**
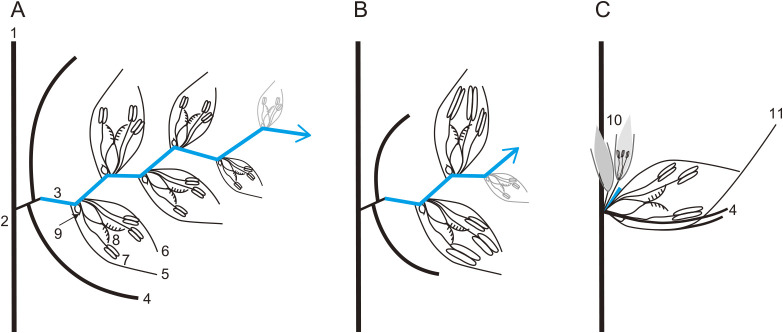
Spikelet determinacy in Triticeae. Cartoons showing wheat (A), rye (B), and barley (C) spikelet features representative of the Triticeae spikelet structure. Spikelets in these species bear florets on the spikelet axis—rachilla (highlighted in blue). In wheat, and rye the indeterminate rachilla bears more than one floret per spikelet whereas in barley the rachilla is determinate and bears a single floret. 1 – Inflorescence meristem; 2 – Rachis; 3 – Rachilla; 4 – Glume; 5 – Lemma; 6 – Palea; 7 – Stamens; 8 – Carpel; 9 – Lodicules; 10 – Lateral spikelets; 11 – Awn.

The branched compound inflorescences of grasses consist of several spikelets as fundamental units. The spikelet structure is unique to grass species which harbors one or more flowers known as florets that are subtended by a pair of bract-like structures called glumes. Typically, the floret of a Triticeae spikelet harbors reproductive organs such as two lodicules, three stamens and a pistil with two styles and stigmas all enclosed between two bract-like structures called lemma and palea (Bonnett 1966, Clayton 1990). Triticeae inflorescences, called “spikes”, differ morphologically from those of other grasses as they have an unbranched simple structure and almost sessile spikelets, meaning their spikelets attach directly to a main axis, the rachis. The Triticeae spike is basically formed by three-levels of meristematic organization, that is the inflorescence meristem producing rachis and spikelet meristems, spikelet meristem producing rachilla, and floret meristem producing florets (Figs. 1, 2). The wheat spike shape is particularly representative of the Triticeae, comprising a single spikelet per rachis node and multiple florets per spikelet (Sakuma *et al.* 2011). The inflorescence (spike) meristem of wheat and its relatives is determinate, with a terminal spikelet at its apex (Fig. 1). The terminal spikelet is formed at the apical end of the rachis, which determines the number of spikelets per spike. Each spikelet generates an indeterminate number of florets attached to a secondary axis, the rachilla. A hexaploid wheat spikelet can produce up to 12 floret primordia; however, generally fewer than four florets survive during development (Guo and Schnurbusch 2015) (Fig. 2). By contrast, the numbers of florets per spikelet in barley are determined to one (Fig. 2). In case of rye, though more florets are formed per spikelet (3–6), always, the first two florets become fertile and bear grains (Fig. 2). The variation in floret number per spikelet within Triticeae species is controlled by the determinate/indeterminate fates of the spikelet axis—rachilla (described in the section “regulation of spikelet determinacy”). The abortion of spikelet primordia or developing florets resulting in sterility is common in the Triticeae species. The lateral spikelets in two-rowed barley and the apical florets in wheat and rye are sterile because their development is suppressed during the growth process. Although the functional and biological significance of the sterile florets remains unknown, several genes that regulate floret development have been elucidated (Koppolu *et al.* 2022a, Sakuma and Schnurbusch 2020).

Recent advances in genome assemblies of the Triticeae species with relatively large genome sizes have accelerated genomics-based research, including the study of developmental biology (Mascher *et al.* 2019). Like rice (*Oryza sativa*), maize (*Zea mays*), and sorghum (*Sorghum bicolor*), the Triticeae are now the subject of extensive genomic analyses (Jayakodi *et al.* 2020, Rabanus-Wallace *et al.* 2021, Walkowiak *et al.* 2020). The genetic diversity of the entire domesticated barley collection (~20,000 accessions) maintained at IPK, the German federal ex situ genebank, has been elucidated (Milner *et al.* 2019). Combining colossal sample sizes and ultra-dense DNA marker data has afforded great power for genome-wide association scans studies (GWAS). Following this, several studies reported population genomic analysis of wheat and rye (Gaurav *et al.* 2022, Sun *et al.* 2022). Importantly, abundant genetic stocks are available from genebanks for research and breeding purposes upon request (Knüpffer 2009, Schulthess *et al.* 2022). Furthermore, several induced mutant populations have been developed, with the TILLING platform being particularly highly efficient and cost effective for generating targeted mutant lines (Jiang *et al.* 2022). The exomes of 2,735 mutant lines of tetraploid and hexaploid wheat have been sequenced, revealing more than 10 million mutations in the protein-coding regions (Krasileva *et al.* 2017). This public collection of mutant wheat stocks enables rapid gene identification and the elucidation of previously hidden variation. Along with genomic datasets, transcriptional data also have been collected for barley and wheat (Pfeifer *et al.* 2014, Thiel *et al.* 2021). These multi-omics data have revolutionized research strategies and accelerated functional genomics in Triticeae species. These resources are all freely available on web-accessible, user-friendly platforms (Ma *et al.* 2021, Zhang *et al.* 2021) listed in Table 1. In addition, advances in genetic transformation and targeted genome modification have impacted Triticeae research (detailed in a review by Hisano *et al.* (2021). In the present review, our current understanding of the genetic basis of Triticeae inflorescences with a focus on barley and wheat, especially the genes controlling 1) spike branching and 2) spikelet/floret development, is described (Table 2).

## Genetic basis of spike/spikelet architecture in the Triticeae

Grass species display a vast array inflorescence architectures, ranging from branched panicle/compound spikes to inflorescences with highly reduced branching, as seen in the spike inflorescences of the Triticeae (Kellogg *et al.* 2013). Within the spike-type inflorescences, the inflorescence branches (as seen in panicle inflorescences) are highly reduced to single spikelets attached to the rachis (Koppolu *et al.* 2022a). In barley, the spikelet ridge meristem formed during the double ridge stage differentiates into three spikelet meristems, giving rise to the canonical triple spikelet meristem (TSM; Koppolu *et al.* 2013) that develops into the triple spikelet structure (three spikelets per rachis node; Fig. 1). By contrast, the spikelet ridge in wheat differentiates into a single spikelet meristem, limiting the number of spikelets per rachis node to one, indicating a further reduction in spike inflorescence complexity within the Triticeae (Bonnett 1935, 1936) (Fig. 1). Canonical Triticeae spikes are therefore devoid of visible long or short lateral inflorescence branches. Another important component of spike architecture within the Triticeae is spikelet determinacy, which dictates the number of florets generated per spikelet. The spikelets of barley are determinate as they bear a single floret on the spikelet axis, known as the rachilla, whereas the wheat spikelets are indeterminate, bearing up to 12 florets on elongated rachillas (Fig. 2). The genetic basis of the spike/spikelet architecture within the Triticeae has been well elucidated over the last 10 years. Various barley and wheat developmental mutants showing perturbations in canonical spike/spikelet architecture (spike branching/spikelet indeterminacy) have been characterized and the underlying genetic and molecular mechanisms were revealed. In the following sections, we elucidate these mechanisms under the subsections (i) Regulation of spike branch outgrowth, (ii) Regulation of supernumerary spikelet formation, and (iii) Regulation of spikelet determinacy.

### Regulation of spike branch outgrowth

**Fig. 3.**
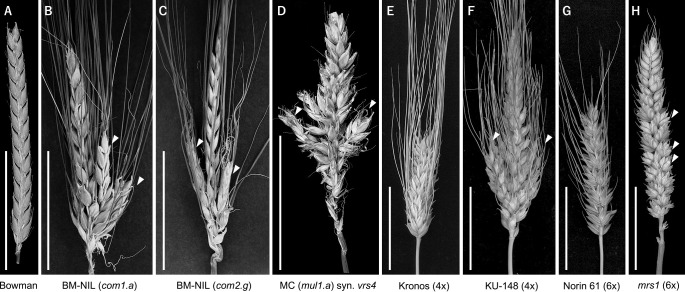
Spike branching regulation in barley and wheat. (A) Unbranched wildtype barley spike (2-rowed). (B–D) mutant spikes of *compositum 1* (*com1*; B), *compositum 2* (*com2*; C), and *six-rowed spike 4* (*vrs4*; D) showing long lateral branches developing from the basal to mid region of the spike. (E) Unbranched tetraploid wheat spike. (F) Branched tetraploid wheat spike. (G) Unbranched hexaploid wheat spike. (H) Multiple-spikelet hexaploid wheat spike. White arrowheads indicate spike branches/supernumerary spikelets. Scale bars = 5 cm.

Within the Triticeae cereals, the active branch suppression and spike inflorescence shape are modulated by a class of genes called *COMPOSITUM*s (*COM*). *COM1* encodes a class II TCP transcription factor (TF), whereas *COM2* (an ortholog of rice *FRIZZY PANICLE*) encodes an AP2-ERF TF (Poursarebani *et al.* 2015, 2020). Mutations in barley *com1* and *com2* result in the loss of spikelet meristem identity, leading to the development of lateral branch-like structures (secondary spikes) in place of spikelets (Fig. 3A–3C). Intriguingly, the barley *com1 com2* double mutants display an enhanced spike-branching phenotype in comparison with the single mutants, indicating the additive nature of these genes towards the branching phenotype (Poursarebani *et al.* 2020). Similarly in tetraploid wheat, *branched head^t^* (*bh^t^*; *TtBH1*), the ortholog of barley *COM2*, functions to suppress spike branching (Poursarebani *et al.* 2015) (Fig. 3E, 3F). Another important regulator of the branch suppression pathway in barley is the row-type gene *SIX-ROWED SPIKE 4* (*VRS4*; *HvRAMOSA2*). Like the *com* mutants, *vrs4* mutants also display a loss of spikelet meristem identity and lateral branch outgrowth (Fig. 3A, 3D) (Poursarebani *et al.* 2015). VRS4 is believed to directly or indirectly regulate the transcription of both *COM1* and *COM2* to modulate the unbranched spike inflorescence shape of barley (Poursarebani *et al.* 2015, 2020). The roles of *COM1* and *VRS4* in wheat spike development are unknown; however, it is interesting to speculate that these genes may play similar roles in suppressing branching to maintain a spike-shaped inflorescence across the Triticeae. Two other wheat spike-branching loci causing false spike ramifications (extended rachilla elongation), *sham ramification 1* and *2* (*shr1*, *shr2*), have been genetically mapped, but their underlying genes are not known (Amagai *et al.* 2014, 2015, Dobrovolskaya *et al.* 2017).

In a recent study, another barley gene, *HvMADS1* (encoding the SEPALLATA TF), was shown to suppress spike branching at high temperatures to maintain a regular spike shape (Li *et al.* 2021a). Under high temperatures, HvMADS1 activates the transcription of genes associated with inflorescence differentiation and phytohormone signaling, especially the gene encoding the cytokinin-degrading enzyme CYTOKININ OXIDASE 3 (HvCKX3), to integrate the temperature response and cytokinin homeostasis, which is required to repress cell division in the meristems (Li *et al.* 2021a).

### Regulation of supernumerary spikelet formation

Archetypal wheat spikes harbor distichously arranged single spikelets at each rachis node, whereas barley spikes harbor three spikelets per rachis node. In both wheat and barley, some spike forms with supernumerary/paired spikelets (SSs; > typical spikelet number per node position) that deviate from the canonical spikelet arrangement exist. These SSs generally form adaxially to the primary spikelet position (Boden *et al.* 2015), and are often referred to as short spike branches.

The genetic and molecular regulation of the SS phenotype has been well characterized in wheat (Fig. 3G, 3H). The first step towards understanding the mechanism of SS formation came from the cloning of *MULTI ROW SPIKE* (wheat *FRIZZY PANICLE*; *WFZP*; encoding an AP2-ERF TF; (Dobrovolskaya *et al.* 2015)). In hexaploid wheat, mutations in the *WFZP-D* homoeolog drive the SS phenotype while the mutations in *WFZP-A* along with *WFZP-D* mutation enhance the SS phenotype (Du *et al.* 2021, Li *et al.* 2021b) (Fig. 3G, 3H). Recently, DUO-B1, yet another AP2-ERF TF, has been implicated in the control of SS formation in wheat. Interestingly, mutants of wheat *DUO-B1* showed an SS phenotype similar to the *multirow spike* mutants, and a further molecular analysis revealed that DUO-B1 suppresses cell division and positively regulates the expression of *WFZP* to control SS development (Wang *et al.* 2022b).

Various other genes have recently been reported to regulate the SS phenotype, including the *PHOTOPERIOD RESPONSE LOCUS1* (*PPD-1*), *FLOWERING LOCUS T 1* (*FT1*), and the major domestication gene *TEOSINTE BRANCHED 1* (*TB1*) (Boden *et al.* 2015, Dixon *et al.* 2018). It has been shown that TB1 and PPD1 regulate FT1—the central regulator of the floral meristem identity gene cascade to control SS formation in wheat. In the photoperiod-insensitive mutant *Ppd-D1*, *FT1* expression is attenuated, promoting SS formation by delaying spikelet meristem maturation (Boden *et al.* 2015). Intriguingly, TB1 also promotes SS formation by attenuating FT1. However, TB1 mediates FT1 attenuation in a manner distinct to *Ppd-1*, where TB1 forms protein complex with FT1. In the gain-of-function wheat *TB1* alleles, the TB1 protein competitively binds to FT1, making it less available to promote meristem maturation (Dixon *et al.* 2018). In another study, Dixon *et al.* (2022) showed that semidominant alleles of the wheat A and D homoeologous genes encoding the class III homeodomain-leucine zipper TF HOMEOBOX DOMAIN-2 (HB-2) promote SS formation. In contrast to the previous mechanisms, the regulation of SS formation by *HB-2* is modulated through microRNA-based regulation; in the semi-dominant alleles of *HB-2*, the complementary *microRNA165/166* (*miR165/166*) binding site is disrupted, leading to elevated levels of *HB-2* transcripts known to promote leaf and vascular development and increase the amino acid supply required for grain development in the SS (Dixon *et al.* 2022).

Various uncharacterized genomic regions controlling SS formation have also been identified through quantitative trait loci (QTL) studies, indicating that SS is a highly quantitative phenotype (Boden *et al.* 2015, Echeverry-Solarte *et al.* 2014, Wolde *et al.* 2021). Despite the wealth of genetic evidence available for the regulation of SS formation in wheat, our genetic knowledge of this phenotype in barley is completely lacking. Although a class of barley mutants called *extrafloret* (*flo.a*, -*.b*, and -*.c*) and another mutant *multiflorus 2.b* display the SS phenotype (Koppolu *et al.* 2022b). However, the genes underlying these mutant phenotypes are not yet known.

### Regulation of spikelet determinacy

Spikelet determinacy in wheat and barley is largely determined by the elongation or suppression of the spikelet axis, known as the rachilla. In the determinate barley spikelet, the rachilla degenerates after producing one floret, whereas in the indeterminate wheat spikelet the rachilla continues to elongate, producing up to 12 florets before being degenerated (Fig. 2). In the majority of grasses, orthologous *APETALA 2* (*AP2*) genes, maize *INDETERMINATE SPIKELET1* (*IDS1*)/*TASSELSEED 6* (Chuck *et al.* 1998, 2007, 2008), rice *IDS1* (Lee and An 2012), and wheat *Q* (*AP2L5*) (Debernardi *et al.* 2017, Greenwood *et al.* 2017) regulate rachilla elongation, thereby controlling the floret number per spikelet. In a recent discovery, barley researchers showed that *HvAP2L-H5* (an ortholog of *Q*) regulates the determinate fate of barley spikelets, with *ap2l5* mutants losing the determinate nature of spikelets and producing more than one floret on elongated rachilla (Zhong *et al.* 2021). Another barley mutant, *multiflorus 2*, produces indeterminate spikelets bearing up to three florets on its elongated rachillas (Koppolu *et al.* 2022b); however, the gene(s) regulating this phenotype are yet to be identified.

Interestingly, in the lateral spikelets of the barley spike-branching mutants *com1*, *com2*, and *vrs4*, the rachilla elongates to produce more than one floret/spikelet, indicating a loss of spikelet determinacy in these lines (Koppolu and Schnurbusch 2019). From these studies, it is evident that the elongation or suppression of the rachilla (the spikelet axis) can dictate the floret number per spikelet, making it an important yield-determining organ in these grass crops.

## Genes regulating spikelet/floret development

The structure and development of the spikelet are key determinants of the grass reproductive organ (Kellogg 2022, Wang *et al.* 2022a). The number of spikelets per rachis node and florets within a spikelet are diagnostic characters of the Triticeae (Sakuma *et al.* 2011). The most common Triticeae spikelet form is a single spikelet per rachis node, as seen in wheat and rye (Bonnett 1966). Unlike the single spikelet type, barley produces a triple-spikelet meristem, resulting in one central spikelet and two lateral spikelets per rachis node. This character is unique to barley and wild *Hordeum* species (Bothmer *et al.* 1995).

The spikelet is distinguished by two glumes surrounding one or more florets; thus, the differentiation of the glume primordia is also a key determinant of spikelet identity. The function of the glumes is not yet well understood; however, their toughness is very important for grain retention or easy threshability in domesticated wheat. During wheat domestication, a dominant mutation at the *Q* locus was fixed during the artificial selection of spikes that were easier to thresh (Simons *et al.* 2006). The expression of *Q*, encoding an AP2-like TF, is negatively regulated by *miR172*; a reduction in *miR172* expression led to higher levels of *Q* expression and greater similarity between glumes and lemmas. Conversely, high levels of *miR172* and the loss of function of *Q* (three homoeoalleles) leads to sterile lemmas and the indeterminacy of the spikelet meristem (Debernardi *et al.* 2017). In the lowermost spikelets, the transition between glumes and lemmas appeared particularly malleable, such that more *miR172* and less *AP2L5* could lead to glume-like organs in the position of lemmas (i.e., sterile lemmas).

Variation in the size and position of the glumes is also evident in Triticeae species, with enlarged distichous glumes in wheat and smaller and more pointed parallel glumes in barley (Fig. 1). Longer glume is particularly evident in the tetraploid wheat *Triticum turgidum* ssp. *polonicum* (also termed *T. polonicum*). Recent studies revealed that the ectopic expression of *VEGETATIVE TO REPRODUCTIVE TRANSITION 2* (*VRT2*, *P1* locus, chromosome 7A) in the spikelet organs underlies the elongated glume phenotype of *T. polonicum* (Adamski *et al.* 2021). The gene encodes a MADS-box TF belonging to the SHORT VEGETATIVE PHASE family. In addition, the paralog of *VRT2* (*SVP-A1*) is proposed to be a candidate gene of the *P2* locus on chromosome 6A (Chen *et al.* 2022). In *Triticum isphanicum*, which develops enlarged glumes, a 482-bp deletion in the *SVP-A1* promoter was found to be associated with the ectopic and higher expression of this gene in the elongated glumes. In barley, the *third outer glume 1* (*trd1*) mutant, is characterized by the outgrowth of leaf-like structures (bracts) in between the two glumes of the central spikelets. The gene underlying *trd1* has been shown to encode the GATA TF, and is orthologous to rice *NECKLEAF 1* (*NL1*) and maize *TASSELSHEATH 1* (*TSH1*) (Houston *et al.* 2012). Interestingly, rice spikelets show rudimentary glumes and empty glumes (called sterile lemmas). The mutation of rice *long sterile lemma1* (*g1*), encoding an ALOG protein, appears to promote the homeotic transformation of the sterile lemma into a lemma-like structure (Yoshida *et al.* 2009). Several studies suggest that the putative *Oryza* ancestor had three florets, with the two lateral florets degenerated during evolution (Ren *et al.* 2013, Yoshida *et al.* 2009).

## Floret abortion/fertility

Floret fertility is the most important trait determining the final grain number of each inflorescence. A single floret is composed of, from the outside, a lemma with or without an awn, a palea, three stamens, two lodicules, and a pistil (Fig. 2). The lemma and palea are considered to be leaf-like structures containing chlorophyll. In Triticeae crops, several key genes regulating floret development have been identified in the last two decades. A six-rowed spike phenotype is a major target for barley researchers, with the row-type determining gene *Vrs1* first cloned as key suppressor of floret fertility at the lateral spikelets (Komatsuda *et al.* 2007). The gene encodes a homeodomain leucine zipper class I TF, which is unique to the plant kingdom. The wheat *Vrs1* ortholog (*Grain Number Increase 1*; *GNI1*) was also found to be a suppressor of apical florets within the spikelets (Sakuma *et al.* 2019). The loss of *Vrs1*/*GNI1* function results in more grains formed per spike. Interestingly, the wheat spikes appear to be evolved to produce more fertile florets per spikelet with the increase in ploidy level and associated heterochrony in floret development (Shitsukawa *et al.* 2009). The diploid einkorn wheats usually set one grain, while the tetraploid wheats set two or three, and the hexaploid wheats set more than three grains per spikelet (Sakuma *et al.* 2019, Shitsukawa *et al.* 2009). It is interesting to speculate that the diploidy in barley and rye could be one of the reasons for lower number of florets/grains formed per spikelet, compared to hexaploid wheat, however research-based evidence is necessary to back this hypothesis. A special allele of barley *Vrs1*, originally endemic to Ethiopia, was also identified (Sakuma *et al.* 2017). The causal mutation of this mutant allele, known as *deficiens*, is a single amino acid substitution located at an unknown C-terminal domain. The *deficiens* spike shows rudimentary lateral spikelets and enlarged grains in the central spikelet. In barley, the induced six-rowed spike mutants *vrs2*, *vrs3*, *vrs4*, and *vrs5* have also been cloned, although these mutant alleles are not yet utilized in breeding programs (Bull *et al.* 2017, Koppolu *et al.* 2013, Ramsay *et al.* 2011, van Esse *et al.* 2017, Youssef *et al.* 2017). The function of the wheat orthologs of *vrs2*, *vrs3*, and *vrs4* remain unknown; however, the wheat *vrs5* ortholog *TB1* was found to be a regulator of paired spikelet formation (Dixon *et al.* 2018). Wheat *TB1* also regulates plant height and the length of the stem internode (Dixon *et al.* 2020).

## Awn development

Awns are needle-like structures, elongated from apex of the lemma in grasses (Fig. 2). Triticeae species present diverse awn morphologies, ranging from long to short awns. Spring-type bread wheat cultivars tend to have long awns, while winter-types have short/tipped awns. Awns in Triticeae crops contribute to photosynthesis and grain yields in warmer and drier rainfed environments (Guo and Schnurbusch 2016, Rebetzke *et al.* 2016). Several studies have attempted to understand awn function, with some researchers hypothesizing that awns use C_4_ photosynthesis rather than the C_3_ photosynthesis typically used by the Triticeae (AuBuchon-Elder *et al.* 2020, Tambussi *et al.* 2021). Awns are also important from the point of view of domestication and adaptation of wild species. Wild emmer wheat characteristically has two long awns per spikelet. The arrangement of cellulose fibrils causes the awns to bend with changes in humidity, helping them to play a role in seed dispersal by either fixing into the soil or attaching to wild animals (Elbaum *et al.* 2007). The genetic basis of awn bending is currently unknown. In wheat, awn elongation is suppressed by three dominant genes, *Tipped1* (*B1*) on chromosome 5A, *Tipped2* (*B2*) on chromosome 6B, and *Hooded* (*Hd*) on chromosome 4A (Yoshioka *et al.* 2017). *B1* was identified as a gene encoding C2H2 zinc finger with ethylene-responsive element binding factor-associated amphiphilic repression (EAR) motifs (Huang *et al.* 2020). Constitutive overexpression of *B1* is responsible for awn inhibition together with pleiotropic effects on plant height and fertility. Haplotype analysis revealed that four SNPs located in *B1* promoter region are associated with awn length, grain length and thousand-grain weight (Wang *et al.* 2020). The investigation of 231 wheat lines from the NIAB MAGIC population found that the presence of awns increased the grain calcium content without decreasing the flour extraction rate, despite the negative correlation between these traits (Fradgley *et al.* 2022).

## Floret opening and its importance towards hybrid breeding

Hybrid breeding has the potential to boost Triticeae crop yields. Indeed, breeding programs for allogamous rye have succeeded in enhancing its inflorescence structure, large anther extrusion, pollen production, and the development of efficient cytoplasmic male sterility (CMS) coupled with nuclear *Restorer-of-fertility* (*Rf*) genes (Miedaner *et al.* 2022, Vendelbo *et al.* 2020). Hybrid seed production in autogamous plants such as wheat and barley remains challenging however, because they require a self-pollination block. Although the molecular mechanisms underlying *Rf* genes have been elucidated in wheat (Melonek *et al.* 2021), the hybrid seed production system is currently insufficient. Understanding floret structure, including the development of the anthers and lodicule, is therefore important. Three anthers are produced in Triticeae florets, there is a correlation between anther length and number of pollen grains. Although the size and number of pollen grains is crucial for the success of hybrid breeding, its genetic basis remains unknown. The *Rht-D1a* allele results in a tall stature in bread wheat, but is also associated with large anthers and a high anther extrusion, despite not enhancing the anther filament length (Okada *et al.* 2021). The lodicule functions to open the lemma and palea during anthesis. Lodicules in cleistogamous barley remain small due to the lack of vascular tissue, a phenotype which is under the control of the *Cleistogamy 1* (*Cly1*) locus encoding an AP2 TF (Nair *et al.* 2009). The cleistogamous barley cultivars possess the *cly1.b* allele, which is distinguished by a synonymous mutation at the *miR172* binding site. This change results in the reduced abundance of the CLY1 protein, but not of its transcript (Anwar *et al.* 2018).

## Grain shattering

Grain shattering has long been recognized to cause yield losses in cereal crops (Bolland 1984, Clarke 1981, Sugimoto *et al.* 2010). Grain shattering is distinguished from the brittle rachis trait shown in wild barley and wild emmer wheat (Pourkheirandish and Komatsuda 2022), with the causal loss-of-function mutations located at *Non-brittle rachis 1* (*btr1*) and *btr2* (Avni *et al.* 2017, Pourkheirandish *et al.* 2015). In grain shattering, spikelet disarticulation from rachis occurs above the glume whereas in brittle rachis phenotype, disarticulation occurs below the glume (Sakuma *et al.* 2011). Some wheat varieties growing in heat-prone and drylands such as Sudan show grain-shattering habits, in which grains fall to the ground when harvesting is delayed. This results in a yield loss of up to 30%; thus, the development of cultivars with reduced shattering while maintaining threshability is an important breeding goal. Recent study by Bokore *et al.* (2022) revealed that a major QTL on chromosome 4BS is associated with reduced grain shattering, and a second QTL was detected on chromosome 5AL. These works shed light on grain shattering resistance and provide DNA markers for developing new cultivars.

The causes and impact of grain shattering (syn. head shattering, head loss) have been reported in barley too (Kandemir *et al.* 2000). A major QTL for head shattering (designated *Hst-3*) has been mapped onto the centromeric region of chromosome 3H, which is different from the *Btr1*/*Btr2* loci. Some barley cultivars drop their spikes onto the ground during a heatwave (Curry and Paynter 2019). In barley, a spontaneous mutant in a cultivar named Kamairazu, which means “sickle not needed to harvest”, has leaves and stems that are easily broken when physically bent (Takahashi *et al.* 1953). This extraordinary fragility is exhibited even after maturity. The locus controlling this fragile phenotype is located on chromosome 5HL, and has been renamed *fragile stem 1* (*fst1*). The identification of the gene underlying the *fst1* and a deeper understanding of spike shattering would be valuable for future cereal breeding under ongoing climate change.

## Future perspectives

As discussed above, inflorescence shape fundamentally contributes to final grain number and size, which is critical to final grain yield. The branched inflorescences in general tend to produce more grains per inflorescence. For example, the panicle inflorescences of rice and sorghum produce more grains per inflorescence compared to species possessing reduced or unbranched inflorescences as in barley and wheat. However, tillering and the grain size trade-offs are evident between branched and unbranched inflorescences. Comparative yield studies across species bearing different inflorescence types can give an understanding about the influence of inflorescence forms on final grain yield in these species. Also, a thorough understanding of the genetic basis underlying these inflorescence shapes helps boosting the yield potential and improve yield stability.

The effects of climate change are already felt around the world, and are expected to worsen significantly over time. Ongoing increases in temperature and rainfall variability in Triticeae crop–growing areas, including temperate, semi-arid, and dryland regions, limit yield improvements, with large reductions and problematic variability in production predicted to occur as a result of climate change. Drylands are a frontier of global warming; hence, understanding crop growth phenomena in drylands is crucial for predicting future patterns of agriculture. Record high temperatures and droughts occur almost every year in relatively stable regions such as Europe, with regular flooding across Japan. To adapt to these climate shifts, the development of new crop varieties using novel strategies and genomic diversity is urgently required. However, our understanding of inflorescence shape and development against such kinds of stress and response is still limited. The first step towards understanding the influence of temperature on spike branch outgrowth regulation has been laid out by characterization of HvMADS1 and its role in maintaining spike shape under ambient temperatures (Li *et al.* 2021a). However, there is still a strong demand for research and investigation for precise understanding of the influence of temperature and water stress towards inflorescence morphogenesis. The outputs from such research activities can ultimately pave the way for the development of climate-resilient cultivars adapted to various environments by leveraging genetic resources and genomic tools.

## Author Contribution Statement

SS conceptualized the review and participated in drafting the initial version, preparation of illustrations and revision of the review. RK participated in drafting the initial version, preparation of illustrations and revision of the review. Both authors have read and agreed upon the final content of the review.

## Figures and Tables

**Table 1.** Useful databases for functional genomics in the Triticeae

Website name	URL
GrainGenes	https://wheat.pw.usda.gov/GG3/
BARLEX	https://apex.ipk-gatersleben.de/apex/f?p=284:10
IPK Galaxy Blast Suite	https://galaxy-web.ipk-gatersleben.de/
Barley Genes and Barley Genetic Stocks	https://www.nordgen.org/bgs/
Barley spike transcriptional landscape	http://bar.utoronto.ca/eplant_barley/
Wheat eFP Browser	http://bar.utoronto.ca/efp_wheat/cgi-bin/efpWeb.cgi
Wheat Expression Browser	http://www.wheat-expression.com/
WheatOmics	http://wheatomics.sdau.edu.cn/
NBRP-wheat	https://shigen.nig.ac.jp/wheat/komugi/
NBRP-barley	http://earth.nig.ac.jp/~dclust/cgi-bin/index.cgi
SeedStor	https://www.seedstor.ac.uk/
Wheat training	http://www.wheat-training.com/

**Table 2.** List of genes discussed in this review

Phenotype	Gene/locus-barley	Gene/locus-wheat	Morex V2 gene ID	Chinese Spring v2.1 gene ID			Protein
Spike branching	*COMPOSITUM 1* (*COM1*)	Unknown function	HORVU.MOREX.r2.5HG0397500.1	TraesCS5A03G0537300.1	TraesCS5B03G0548700.1	TraesCS5D03G0500500.1	class II CYC/TB1 TCP TF
	*COMPOSITUM 2* (*COM2*)	*branched head^t^*	HORVU.MOREX.r2.2HG0093930.1	TraesCS2A03G0239400.1	TraesCS2B03G0329100.1	TraesCS2D03G0248500.1	AP2-ERF TF
	*Six-rowed spike 4* (*vrs4*)	Unknown function	HORVU.MOREX.r2.3HG0194160.1	TraesCS3A03G0206100.1	TraesCS3B03G0249000.1	TraesCS3D03G0184600.1	LOB domain TF
	*HvMADS1*	Unknown function	HORVU.MOREX.r2.4HG0329790.1	TraesCS4A03G0052800.1	TraesCS4B03G0666300.1	TraesCS4D03G0586600.1	SEPALLATA TF
Supernumerary spikelets	*COMPOSITUM 2* (*COM2*)	*MULTI ROW SPIKE* (*MRS*)	HORVU.MOREX.r2.2HG0093930.1	TraesCS2A03G0239400.1	TraesCS2B03G0329100.1	TraesCS2D03G0248500.1	AP2-ERF TF
	Unknown function	*DUO-B1*	HORVU.MOREX.r2.1HG0055140.1	TraesCS1A03G0780000.1	TraesCS1B03G0895200.1	TraesCS1D03G0747200.1	AP2-ERF TF
	*PHOTOPERIOD-H1* (*Ppd-H1*)	*PHOTOPERIOD RESPONSE LOCUS1* (*PPD1*)	HORVU.MOREX.r2.2HG0088300.1	TraesCS2A03G0159800.1	TraesCS2B03G0222600.1	TraesCS2D03G0156800.1	PSEUDO RESPONSE REGULATOR (PRR)
	*FLOWERING LOCUS T1* (*FT1*)	*FLOWERING LOCUS T1* (*FT1*)	HORVU.MOREX.r2.7HG0542540.1	TraesCS7A03G0272100.1	TraesCS7B03G0031800.1	TraesCS7D03G0250300.1	Phosphatidylethanolamine-binding protein (PEBP)
	*Intermedium spike-C* (*vrs5*)	*TEOSINTE BRANCHED 1* (*TB1*)	HORVU.MOREX.r2.4HG0280760.1	TraesCS4A03G0702300.1	TraesCS4B03G0092100.1	TraesCS4D03G0066700.1	class II TCP TF
	Unknown function	*HOMEOBOX DOMAIN-2* (*HB2*)	HORVU.MOREX.r2.1HG0033880.1	TraesCS1A03G0425300.1	TraesCS1B03G0515400.1	TraesCS1D03G0402600.1	class III homeodomain-leucine zipper TF
	*EXTRAFLORET* (*FLO*)	Unknown	not yet cloned				—
Spike/spikelet determinacy	*HvAP2L-H5*	*Q*	HORVU.MOREX.r2.5HG0437030.1	TraesCS5A03G1116700.1	TraesCS5B03G1184600.1	TraesCS5D03G1069300.1	APETALA 2-like TF
	*MULTIFLORUS 2* (*MUL2*)	Unknown	not yet cloned				—
Floret development	Unknown function	*VEGETATIVE TO REPRODUCTIVE TRANSITION 2* (*VRT2*)	HORVU.MOREX.r2.7HG0551090.1	TraesCS7A03G0411400.1	TraesCS7B03G0215800.1	TraesCS7D03G0398300.1	MADS-box TF
	*third outer glume 1* (*trd1*)	Unknown function	HORVU.MOREX.r2.1HG0075620.1	TraesCS1A03G1020200.1	TraesCS1B03G1202900.1	TraesCS1D03G0980700.1	GATA TF
	*Six-rowed spike 1* (*vrs1*)	*Grain Number Increase 1* (*GNI1*)	HORVU.MOREX.r2.2HG0152930.1	TraesCSU03G0009700.1	TraesCS2B03G1031100.1	TraesCS2D03G0878100.1	class I homeodomain-leucine zipper TF
	*Six-rowed spike 2* (*vrs2*)	Unknown function	HORVU.MOREX.r2.5HG0413830.1	TraesCS5A03G0773600.1	TraesCS5B03G0808200.1	TraesCS5D03G0732800.1	SHORT INTERNODES (SHI) TF
	*Six-rowed spike 3* (*vrs3*)	Unknown function	HORVU.MOREX.r2.1HG0041980.1	TraesCS1A03G0526300.1	TraesCS1B03G0615000.1	TraesCS1D03G0507600.1	Jumonji C-type H3K9me2/me3 demethylase
	Unknown function	*Tipped1* (*B1*)	HORVU.MOREX.r2.2HG0141060.1	TraesCS5A03G1268100.1	TraesCS4B03G0896100.1	TraesCS4D03G0789800.1	C2H2-type zinc finger TF
	*Cleistogamy 1* (*Cly1*)	*TaAP2*	HORVU.MOREX.r2.2HG0170380.1	TraesCS2A03G1196100.1	TraesCS2B03G1363800.1	TraesCS2D03G1150300.1	APETALA 2 TF
